# A Genome-Wide Association Study Reveals Candidate Genes Related to Salt Tolerance in Rice (*Oryza sativa*) at the Germination Stage

**DOI:** 10.3390/ijms19103145

**Published:** 2018-10-12

**Authors:** Jie Yu, Weiguo Zhao, Wei Tong, Qiang He, Min-Young Yoon, Feng-Peng Li, Buung Choi, Eun-Beom Heo, Kyu-Won Kim, Yong-Jin Park

**Affiliations:** 1 Department of Plant Resources, College of Industrial Sciences, Kongju National University, Yesan 32439, Korea; agnesyu121@ahau.edu.cn (J.Y.); wgzsri@126.com (W.Z.); wtong@ahau.edu.cn (W.T.); qiangh06@gmail.com (Q.H.); myyoon0721@gmail.com (M.-Y.Y.); lifengpeng2013@gmail.com (F.-P.L.); pckorea1587@gmail.com (B.C.); hueunbum@gmail.com (E.-B.H.); 2 State Key Laboratory of Tea Plant Biology and Utilization, Anhui Agricultural University, Hefei 230036, China; 3 School of Biotechnology, Jiangsu University of Science and Technology, Sibaidu, Zhenjiang, Jiangsu 212018, China; 4 National Key Facility for Crop Resources and Genetic Improvement, Institute of Crop Science, Chinese Academy of Agricultural Sciences, Beijing 100081, China; 5 Leader of Eco. Energy & Bio (LEEBCOR), 190-26 Hwangyeonggongwon-ro, Asan-si, Chungcheongnam-do 31529, Korea; 6 Suzhou GENEWIZ Biotechnology Co. LTD, C3 218 Xinghu Road Suzhou Industrial Park, Suzhou 215123, China; 7 Chemical Safety Division, National Institute of Agricultural Sciences (NIAS), Wanju 55365, Korea; 8 Breeding & Research Institute, Koregon Co. LTD, Anseong Center 60-34, Gokcheon-gil, Bogae-Myeon, Anseong-Si, Gyeonggi-Do 17509, Korea; 9 Center of Crop Breeding on Omics and Artificial Intelligence, Kongju National University, Yesan 32439, Korea

**Keywords:** rice, genome-wide association study, salt stress, germination, natural variation

## Abstract

Salt toxicity is the major factor limiting crop productivity in saline soils. In this paper, 295 accessions including a heuristic core set (137 accessions) and 158 bred varieties were re-sequenced and ~1.65 million SNPs/indels were used to perform a genome-wide association study (GWAS) of salt-tolerance-related phenotypes in rice during the germination stage. A total of 12 associated peaks distributed on seven chromosomes using a compressed mixed linear model were detected. Determined by linkage disequilibrium (LD) blocks analysis, we finally obtained a total of 79 candidate genes. By detecting the highly associated variations located inside the genic region that overlapped with the results of LD block analysis, we characterized 17 genes that may contribute to salt tolerance during the seed germination stage. At the same time, we conducted a haplotype analysis of the genes with functional variations together with phenotypic correlation and orthologous sequence analyses. Among these genes, *OsMADS31*, which is a MADS-box family transcription factor, had a down-regulated expression under the salt condition and it was predicted to be involved in the salt tolerance at the rice germination stage. Our study revealed some novel candidate genes and their substantial natural variations in the rice genome at the germination stage. The GWAS in rice at the germination stage would provide important resources for molecular breeding and functional analysis of the salt tolerance during rice germination.

## 1. Introduction

Salt stress undesirably affects plant growth during all developmental stages. Therefore, it is a major threat to crop productivity [1] and this situation has lasted in some parts of the world for over 3000 years with growth every year. As a monocotyledonous model plants, rice feeds more than one half of the world’s population [2]. However, rice is also sensitive to salt stress and is currently listed as the most salt-sensitive cereal crop, which results in most cultivated varieties having a salinity threshold of 3 dSm^−1^ [3].

Seed germination is usually a very important stage in the seedling stable stand establishment and determines the success of crop production [4]. The effects of salt stress on seed germination are extremely complex involving various physical and biochemical cues. Generally, salt stress is negatively correlated with seed germination and seedling growth [5] in most plants such as *Oryza sativa* [6], *Zea mays* [7], *Helianthus annuus* [8], and *Brassica* spp. [9]. The impact of salt stress on seed germination is attributed to seed water uptake and ion toxic effect. During the seed germination, salinity alters the imbibition of water by reducing the osmotic potential of the germination medium [10], damages the ultrastructure of cells, tissue, and organs [11], changes the activity of enzymes [12], disturbs hormonal balance [13], alters protein metabolism [14], and reduces the use of seed reserves [15]. However, various environmental (external) and plant physiological (internal) factors affect seed germination under saline conditions including temperature, light, water and gasses, seed age, seed dormancy, nature of seed coat, seed morphology, and seedling vigor [16].

Recently, improving rice salt tolerance during the germination stage became more important because salinity may rapidly reduce the germination rate and percentage, which, in turn, may lead to a reduction of crop yields [17]. Many efforts have been made to improve seed germination and seedling vigor by optimizing the non-genetic factors [6]. However, success in improving salt tolerance in rice is made by identifying a major quantitative trait locus (QTL), which contributes to salt tolerance in rice. QTL analysis of seed germination have been reported in rice [18], soybean [19], wheat [20], *Arabidopsis* [21], and *Brassica rapa* [22]. However, it is difficult to develop rice elite varieties with a high level of salt tolerance due to a lack of understanding the mechanisms of salt tolerance during the seed germination stage. Moreover, QTLs conferring salt stress tolerance in rice were identified mainly at the seedling stage [23], but there are few reports on rice seed germination [18,24,25]. Wang et al. [18] detected 16 QTLs for the imbibition rate and germination percentage under control and salt stress with the recombinant inbred line (RIL) population derived from IR26/Jiucaiqing. Abe et al. [26] identified a candidate gene, *OsGA20ox1*, for a major QTL controlling seedling vigor in rice. Zheng et al. [27] identified 11 QTLs for salt tolerance at the germination and early seedling stage in *japonica* rice.

Genome-wide association study (GWAS) is an efficient method for detecting valuable natural variations in trait-associated loci as well as allelic variations in candidate genes underlying quantitative and complex traits [28,29]. Instead of SSR (simple sequence repeat) markers, which are commonly used for association mapping [30,31], SNPs (single-nucleotide polymorphisms) have become more popular for GWASs with the rapid development of NGS (next-generation sequencing) and the existence of high-density SNP markers by re-sequencing [32]. In rice, there are some successful reports to dissect genetic architecture of complex traits through GWAS [28,29,32,33]. However, limited studies have been carried out in rice to identify genes/QTLs for salt tolerance using GWAS. Kumar et al. [34] identified total 64 SNPs significantly associated with Na^+^/K^+^ ratio and other traits for reproductive stage salinity tolerance using GWAS. Yu et al. [35] identified 93 candidate genes significantly associated with salt tolerance at the rice seedling stage. Shi et al. [36] identified 22 significant salt tolerance associated SNPs based on the stress-susceptibility indices (SSIs) of vigor index (VI) and the mean germination time (MGT). Naveed et al. [37] identified 20 QTN for salinity tolerance at the germination and seedling stages in rice. Seed germination plays an important role in the cycle of plant growth. To our knowledge, there is little research until now on the identification of genes particularly for the germination stage salinity tolerance using GWAS in rice. Here, we applied GWAS mapping using ~1.65 million SNPs/indels covering all 12 rice chromosomes in a diverse rice collection to identify candidate genes and natural variation that may contribute to salt tolerance during the rice germination stage with the aim to guide breeding of salt-tolerant rice varieties.

## 2. Results

### 2.1. Phenotypic Screening and Evaluation

**Figure 1:**
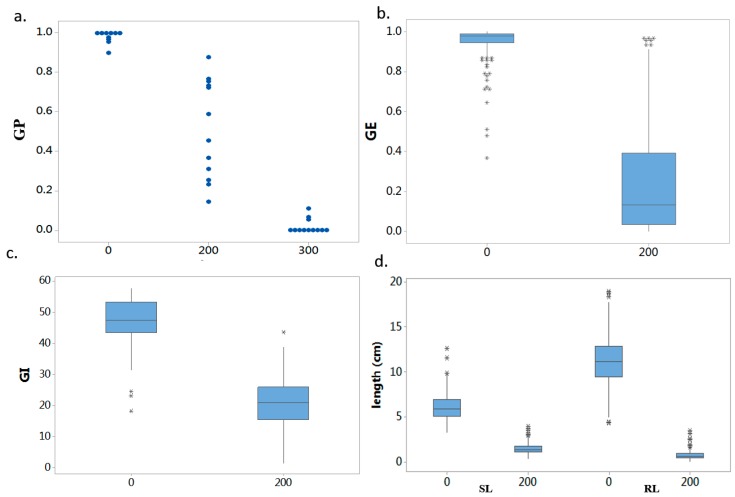
Determination of the optimum NaCl concentration and main phenotypes under salt stress and control conditions. (**a**) Individual value plot of germination percentage in the presence of 0, 200, and 300 mM NaCl (each dot represents an individual). (**b**–**d**) Box plots for phenotypic values in the presence of 0 and 200 mM NaCl (the asterisks are extreme outliers). GP: germination percentage, GE: germination energy, GI: Germination index, SL: shoot length, RL: root length.

Individual value plots for GP with 0, 200 and 300 mM NaCl from a screening experiment using 12 randomly selected samples are shown in Figure 1a. According to the screening result, 200 mM NaCl fully exhibited their phenotypic variance, which resulted in the most diverse phenotypic distribution and facilitated discrimination of accessions with different salt tolerance levels. Thus, the treatment of 200 mM NaCl was chosen as the target salinity level for determining the salt tolerance of all accessions.

The following traits: GP, GE, GI, SL, and RL were examined under 0 and 200 mM NaCl salt stress during the rice germination stage. Descriptive statistics of the phenotypes related to salt tolerance during the germination stage of the current collection were presented in Table 1. Box plots of phenotypes including GE, GI, SL, and RL in the presence of 0 and 200 mM NaCl are shown in Figure 1b–d. The data suggested that seed germination traits were negatively influenced by salt stress. Salt stress inhibits shoot and root elongation dramatically but GE and RL were more affected than SL [18]. These findings indicated that most germination parameters under salt stress exhibited lower performance than under control conditions, which may restrict plant growth.

The correlation coefficients of phenotypes under control and salt-stress conditions were also evaluated (Table 2). RL was significantly and positively correlated with SL only under control conditions. Excepting RL and SL was also significant positively correlated with GI. GP, GE, and GI were all significantly and positively correlated with each other under control conditions while all phenotypes were significantly and positively correlated with each other in salt stress conditions. These results suggested that all phenotypes evaluated in this study could be used for GWAS and some overlapped results could be found among the phenotypes.

### 2.2. Principal Components Analysis (PCA)

**Figure 2:**
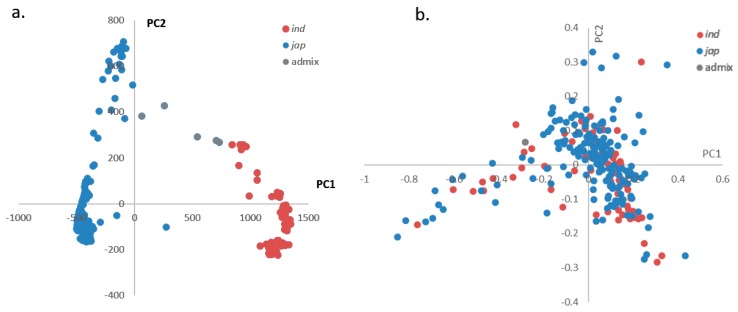
Principal components analysis (PCA) using genotype and phenotype data. (**a**). For genotype data, 295 accessions were divided into ind (*indica*) and jap (*japonica*) based on PC1 and PC2 along with an admixture group. (**b**). For phenotype data, no clear grouping was observed.

PCA was performed with the 1.65 million high-quality SNPs/indels to mine the population structure in all rice accessions. Two components were suggested by the scree plot (Supplementary file 2: Figure S1a). Clear subpopulation structures were observed based on the first two PCs (PC1 and PC2), which resulted in two subpopulations, *indica* and *japonica*, with the admixture accessions located between the two groups (Figure 2a).

For the PCA using the phenotypes, we examined correlations between subspecies in salt tolerance levels using four main phenotypes that drove the differences among accessions. We used TASSEL to perform a PCA of R-GE, R-GI, R-RL, and R-SL in the rice collection. Most of the phenotypic variation (>91%) in the collection was explained by the first two PCs (Supplementary file 1: Figure S1b). Thus, we generated a PCA plot using PC1 and PC2. However, rice accessions in our study were not clustered into clearly defined groups (such as *indica* or *japonica*) based on the above four phenotypes (Figure 2b). This indicates that salt tolerance levels in rice (*O. sativa*) are not strongly correlated with the *indica* or *japonica* subgroups.

### 2.3. GWAS and Candidate Gene Identification

**Figure 3:**
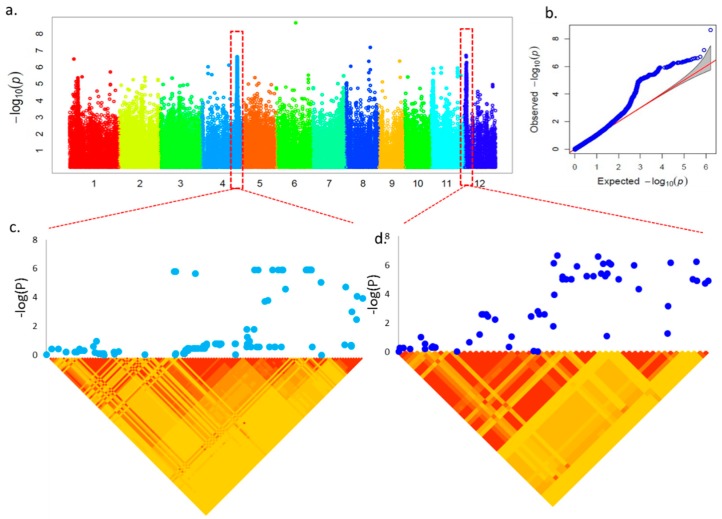
Genome-wide association mapping and LD block analysis for root length (RL) under salt stress (200 mM) conditions. (**a**) Manhattan plot from association mapping using the CMLM. (**b**) QQ plot of expected and observed P values. (**c**) The peak region on chromosome 4 along with the LD blocks. (**d**) The peak region on chromosome 12 along with the LD blocks. In (**c**,**d**) pair-wise LD between SNPs is indicated as *D*’ values: red indicates a value of 1 and yellow indicates 0. The LD region was 200 kb upstream and downstream of the top −log (*p*) value in the peak range.

To generate the genotype dataset for GWAS, more than ~1.65 million SNPs/indels were identified across the accessions and subjected to GWAS applied with the CMLM [38]. The GWAS results were shown on Figure 3 and Supplementary file 1: Figure S2. We took associations held by the peaks with −log10 (*p*) value > 5 and adjusted *p*-value (FDR, false discovery rate) < 0.05 for further analysis since the cutoff of −log10 (*p*) value was five when the FDR ≤ 0.05. Under the salt stress condition, the significant signals were detected for GE, RL, SL, R/S, and R-R/S. In total, 10 SNPs were found significant for these traits and only one SNP (chr12_1628276) were found common for RL, R/S, and R-R/S traits. Excluding the common one, only 3, 2, 2, 1, and 1 SNPs were found uniquely associated for R-R/S, GE, RL, SL, and R/S, respectively. In addition, we also found two SNPs (chr02_1090174 and chr05_20164893) were the two strongest, significantly associated for GE in all observed traits (*p* < 10^−9^).

We further conducted a genome-wide LD analysis of the candidate peak regions and determined LD blocks harboring significant SNPs/indels that characterized in the last step as regions containing putative candidate genes. LD block analysis was detected in a 400 kb range centered on the highest −log10 (*p*) value (Figure 3c,d). Annotation of SNPs/indels from the 200 kb up-stream and down-stream ranges, together with the LD block analysis, resulted in the identification of 79 genes included in these peaks and some candidate genes have been reported previously to contribute to salt tolerance (Supplementary file 2: Table S2). Among the known genes, seven were associated with chromosome 1 (all in RL), 22 with chromosome 2 (7 in GE, 6 in R/S and 9 in R-R/S), 10 with chromosome 3 with R-R/S, 15 with chromosome 4 (3 in SL and 12 in RL), 6 with chromosome 5 (all in GE), 6 with chromosome 11 (all in R-R/S), and 13 with chromosome 12 (the peak region was identical in RL, R/S, and R-R/S). In the LD block analysis, most highly associated SNPs/indels were located in small or large LD blocks, which indicated that they were in significant linkage disequilibrium. Thus, these candidate genes may contribute to salt stress independently or co-operatively with other variations in other genes harboring these SNPs/indels. Simultaneously, we screened candidate genes containing many highly associated SNPs/indels in the genic region as well as some highly associated signals not located in known genes but suggesting that these unknown genes may also be related to salt tolerance (Supplementary file 4: Table S3). Some of those SNPs/indels were located in the coding region of the unknown genes rather than in the surrounding 200 kb regions. These genes could also be important determinants of salt tolerance in rice.

### 2.4. Natural Variations in Candidate Genes and Sequence Analysis

Based on the associated peaks identified in the GWAS and by determining the LD blocks test, we identified many candidate genes associated with salt tolerance during the rice germination stage. To mine functional and novel candidate genes, we investigated 17 final candidate genes (Table 3) that contained highly associated SNPs/indels within the coding region. Many of these SNPs/indels have been reported to play a role in the salt stress in rice such as *OsAGO2* (Os04g0615700) [39], *OsZIFL13* (Os12g0133300) [40], and *OsHAK11* (Os04g0613900) [41], which are related to salt stress in rice. These genes are involved in the salt tolerance in rice by different pathways [42].

**Figure 4:**
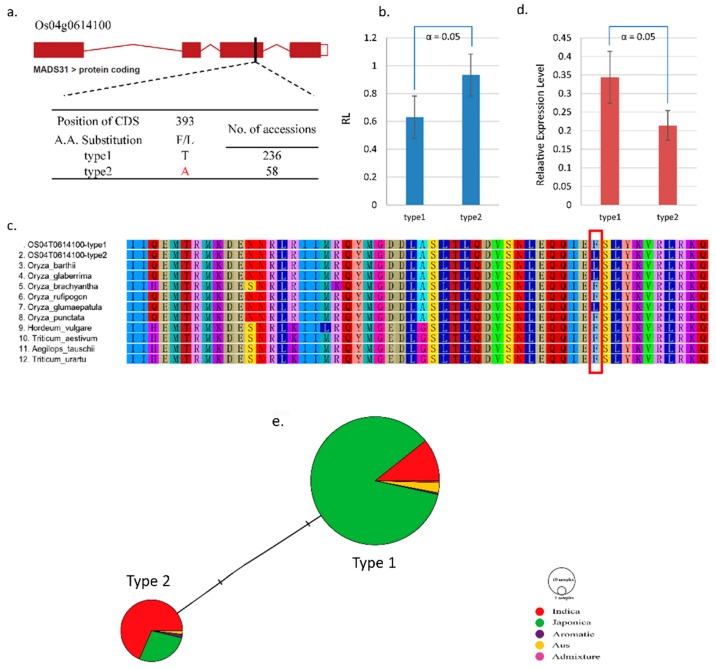
Haplotyping and sequence analysis of Os04g0614100, which was correlated with a phenotypic difference. (**a**) One functional SNP of the candidate gene in the CDS region. Type 1 is the reference and type 2 is the SNP. (**b**) The phenotypic difference based of the functional SNP. (**c**) Amino acid sequence alignment using several orthologues in various rice subgroups and species. Red box indicates the target amino acid change caused by the functional SNP. (**d**) RNA expression levels in rice accessions with type 1 and type 2. (**e**) Haplotype network analysis. Circle size is proportional to the number of samples within a given haplotype. Lines between haplotypes represent mutational steps between alleles. Colors denote rice designation.

Natural variations of these 17 genes were mined and then functional variations were screened after checking the positions of the variations in genes and the corresponding amino acid change. Among the genes, *OsMADS31*, which is involved in floral organ specification and implicated in plant growth and development, was identified and predicted to be involved in salt tolerance [43]. As shown in Figure 4, one natural SNP substitution (T/A) was detected and caused an F/L amino acid change, which is presented by type 1 (reference sequence) and type 2 (variation) (Figure 4a). Furthermore, we generated a haplotype network of the whole collection, which was dominated by two common haplotypes including primarily the *japonica* type (type 1) and the *indica* type (type 2), respectively (Figure 4e). A phenotypic difference was observed in type 1 with 236 accessions and an average RL of 0.6293 and type 2 with 58 accessions and an RL of 0.9327 (Figure 4b). We conducted further orthologue alignment of *OsMADS31* in several rice groups and other species (Figure 4c). Type 2 (candidate SNP) showed an F/L amino acid change compared to other rice groups and species (type 1, *Oryza brachyantha*, *Oryza rufipogon*, *Oryza punctate*, *Hordeum vulgare*, *Triticum aestivum*, *Aegilops tauschii*, and *Triticum urartu*). However, type 2 shared this F/L with three other rice species (*Oryza glaberrima*, *Oryza barthii* and *Oryza glumaepatula*). *Oryza glaberrima* and *Oryza barthii* are African rice and its wild type have higher salt tolerance than *Oryza sativa* species. *Oryza glumaepatula* is a wild rice found in South America usually in deep and sometimes flowing water, which may also have salt tolerance characteristics based on the presence of related genes. Four salt tolerant accessions with type 2 haplotype and four salt sensitive accessions without the haplotype were used for the real-time expression analysis. Generally, the relative RNA expression level of Os*MADS31* was higher in type 1 than in type 2, which indicates that the gene expression is down-regulated in salt conditions when compared to the control (Figure 4d).

We also found several other functional SNPs/indels in the 17 candidate genes that were correlated with a phenotypic difference (Supplementary file 5: Table S4). These candidate genes may be related to rice salt tolerance, according to both previous reports and the natural variation mining in the current study. Novel polymorphisms of those genes may also contribute to salt tolerance that make the rice resistant to salinity.

## 3. Discussion

### 3.1. Salt Tolerance at Rice Germination Stage

The seed germination is one of the most critical steps in the life cycle of a crop. Seed germination begins with water uptake while salinity prevents water imbibition, which inhibits seed germination [15]. Experiments have shown that increased salinity delays the initiation of germination, which leads to a reduced germination percentage. However, salt tolerance during the early growth stages is not always correlated during subsequent growth stages [44,45]. The seeds of crops in different genotypes may germinate adequately under salt stress. However, the seedling may not become fully established later. We observed differential inhibition of the root length and shoot length in our study, which suggested that salinity can influence the germination quality of the seed.

By using the optimized salinity (200 mM NaCl) for discriminating accessions with different salt tolerance levels, we characterized the salt-tolerance-related phenotypes in a collection comprising 295 rice varieties. Phenotypic differences between the control and 200 mM NaCl salinity conditions suggested that rice growth during the seed germination stage can be markedly inhibited by salt stress, which results in very low germination energy and index (GE and GI) as well as reduced root and shoot lengths. This may suppress rice seed germination especially in some direct-sowing areas and decreases plant density and yield markedly. Therefore, development of rice varieties with salt-tolerant seeds would prevent salinity-mediated plant and yield loss during the early growth stage.

### 3.2. Salt Tolerance Is Not Strongly Correlated with Rice Subgroups

According to Lee et al. [46], the salt tolerance of *indica* rice was higher than that of *japonica* rice at the seedling stage, which was determined by measuring shoot Na^+^ and K^+^ absorption. However, as revealed in a recent study of the salt tolerance of 115 *O. sativa* and *O. glaberrima* accessions, salt tolerance was not strongly correlated with *O. sativa* cultivar groups [35]. Most of the *japonica* types were salt sensitive, but accessions from the *indica* group and *O. glaberrima* showed a wide range of sensitivities [47]. In our study, we performed a PCA of all rice germplasm using both genotype and phenotype data. Inconsistent with the genotypic PCA, which separated the collection into clear groups, phenotypic PCA using germination-related phenotypes showed no clear grouping (Figure 2b). This indicated that salt tolerance levels during the seed germination stage are not well correlated with the rice (*O. sativa*) subgroup.

### 3.3. GWAS and Candidate Gene Identification

In some direct-sowing areas, salt tolerance in rice during the seed germination stage is particularly important. To improve rice productivity in such areas, novel genes and alleles associated with complex quantitative salinity tolerance traits must be identified in diverse rice accessions and salt-tolerant varieties bred. An alternate and complementary approach is GWAS, which takes advantage of historical recombination events and, thus, enables a high-resolution genome wide mapping for the identification of target genomic regions in response to complex quantitative traits in rice [29]. In this study, we used a core set of rice collections and multiple bred varieties to investigate candidate loci and genes that regulate important phenotypes under salt stress in rice at the germination stage. Twelve GWAS peaks representing new QTLs on chromosomes 1, 2, 3, 4, 5, 11, and 12 during the rice germination stage were identified. The current association mapping can serve as source of novel salt tolerance genes and alleles. Thus, we found abundant candidate regions with high association peaks in five traits and were distributed on seven chromosomes. Now many QTL analysis of rice salt tolerance have been reported, but it is difficult to directly compare the chromosomal location of marker–trait associations detected in this study with previously reported QTLs because different materials at different stages, descriptive traits, and molecular maps have been used. Wang et al. [18] detected 16 QTLs for the imbibition rate and the germination percentage. Kumar et al. [34] identified 64 SNPs (loci) significantly associated with salt stress-related traits by GWAS. Leon et al. [48] identified 85 additive QTLs for seedling salinity tolerance by GBS. Yu et al. [35] identified 25 SNPs (loci) significantly associated with salt stress-related traits by GWAS. Shi et al. [36] identified 22 SNPs based on SSIs of VI and MGT by GWAS. In this study, we also found that some SNPs associated with salt-tolerance traits overlapped or located in similar or proximal regions such as a significant SNP (chr04_31168058) near *qRTL4.10* identified by Leon et al. [48] and SNPs (chr04_31164404) identified by Yu et al. (2017). This SNP is also located near the SNPs (chr04_34164920 and chr04_ 34292214) identified by Kumar et al. [34] associated with Na^+^/K^+^ ratio. Additionally, two QTLs for salt tolerance and potassium concentration were mapped just prior to this region, respectively, by Lin et al. [49] and Cai and Morishima [50]. The above results also indicated that chromosome 4 including many candidate genes in this region was found to be important for salt tolerance.

So far, about 70 salt tolerance QTLs had been located in rice using biparental mapping populations, but fine mapping and narrowing down reports are limited [34]. Driven by LD blocks to define the genomic regions for searching candidate genes has advantages over the fixed-window approach in which a certain distance from a significant SNP is considered to be the region containing candidate genes [51] by eliminating falsely included or excluded genes [52]. The wide candidate regions ranged from <1 kb to >1 Mb depending on the chromosomal position, which suggests that the resolution of the association mapping is highly dependent on the LD of the neighboring regions of the significant SNPs [34]. Since some of the LD blocks harboring significant SNPs did not contain an annotated gene, this method might have produced some false negatives or the identified region may have contained important DNA-binding or gene regulation sites, in which case, the causal gene was not detected in the LD block [53]. In this work, we used a 400 kb range of the strongest signal to locate the candidate genes, which is in line with previous studies [54]. From an LD block analysis, we obtained 79 candidate genes that had significant SNP/indel associations in LD block regions. Therefore, these regions and candidate genes have a statistically and genetically supported background and, therefore, may be important for the salt tolerance of rice during the germination stage. Apart from SNPs that had an association with previous known QTLs for salinity tolerance, there were a few SNPs, which hit specific genes that were known or functionally characterized for salt stress. Among 79 candidate genes including seven protein kinases (PK) (1 Serine/threonine protein kinase and 1 *OsCDPK26*), six ion exchanger and transporter related genes, five transcription factors (TFs), two electron carrier (peroxidase, Os01g0172600, and oxidoreductase, Os05g0411200), and two major facilitator superfamily proteins (Os12g0133100, *OsZIFL12*; Os12g0133300, *OsZIFL13*). In addition, we also found one stress-associated protein 18 (SAPs) (*OsSAP18*, Os02g0121600), one vacuolar ATPase assembly integral membrane protein (Os04g0612900), two argonaute family proteins (AGOs) (*OsAGO2*, Os04g0615700, and *OsAGO3*, Os04g0615800), one chloroplast precursor (*Ferritin1*, Os11g0106700), one calmodulin-like protein 3 (*OsCML3*, Os12g0132300), one Auxin efflux carrier protein (*OsPIN1d*, Os12g0133800), one Glycoside hydrolase (Os02g0532900), and one Glycosyltransferase (*ALG3*, Os01g0172000). The above results indicated that the candidate genes may play an essential role in salt tolerance mechanisms [34], which also indicated that salt tolerance genes are involved in ion pumps, calcium, the salt overly sensitive (SOS) pathway, mitogen-activated protein kinases (MAPK), glycine betaine, proline, and the reactive oxygen species pathways in a high salinity environment [42].

Furthermore, 17 candidate genes with high −log10 (*p*) value-associated signals inside the coding region were also mined and may play an important role in salt tolerance. *OsHAKs* are candidates for high-affinity K^+^ uptake transporters in the rice root. The transcription of *OsHAK11* (Os04g0613900) is significantly induced by salt stress and K^+^ starvation, respectively [55]. *AGOs* (Os04g0615700) play important roles in the regulation of development and stress responses, antiviral immune response, transposons, and the regulation of chromatin structure and can affect the growth and development as well as the response to abiotic and biotic stress [56]. *OsPIN* (Os12g0133800), which encodes a member of the auxin efflux carrier proteins, is involved in the root elongation growth and lateral root formation patterns via the regulation of auxin distribution in rice [57]. The Germin family protein (Os02g0532500) had been revealed to be connected with a plant cell defense and diseases and to be highly resistant to sodium dodecyl sulfate (SDS) and proteases and important for early plant development and germination in plants [58]. *SAP* (Os12g0133700) is the A20/AN1 zinc-finger containing proteins, which can regulate the stress signaling in plants [59]. The Zinc-induced facilitator-like (ZIFL) family genes (Os12g0133100, *OsZIFL12*, Os12g0133300, *OsZIFL13*) are up-regulated under stress conditions [40].

Based on these regions and candidate genes, it may be possible to mine the natural variations of rice in response to salt stress in some tolerant accessions and apply those alleles to sensitive accessions via breeding methods. To the best of our knowledge, this is the first large-scale GWAS focusing on salt stress during the rice germination stage. These candidate regions and genes will facilitate the development of salt-tolerant rice varieties.

### 3.4. Novel Natural Variations of Candidate Genes

Investigation of new natural variations in focal traits can extend the tolerant varieties’ functional alleles to other non-tolerant varieties. Breeding methods can then be used to transfer them to elite lines to produce tolerant varieties. Using the results of the GWAS and LD analysis, the haplotypes of candidate genes can be targeted and the functional alleles involved in responses can be identified. New alleles in rice have been reported [60,61] and provide insight for researchers and breeders into the underlying mechanisms, which facilitates the breeding of improved varieties. According to Arora et al. [42], *OsMADS31* expression was low and not markedly affected by salt and cold stress. However, the expression was relatively down-regulated in seedlings under a salt condition. Nevertheless, *OsMADS31* expression was higher in seeds than during the panicle stage. In this study, we found that *OsMADS31* was associated with salt tolerance in rice at the seed germination stage with a down-regulated expression in the salt condition (Figure 4d). The contribution of MADS-box genes to flower organ specification is well developed in eudicots, but not very well in rice. Therefore, the roles of MADS genes and other candidate genes identified here using GWAS at the seed germination stage in response to salt stress should be investigated further. Moreover, by adapting functional studies (such as those performed using TALEN and CRISPR/Cas 9), the functions of genes and gene variations can be determined. Natural variations that have functional signals could be a good starting point for the exploration of gene-based assays of phenotypically different individuals such as salt tolerant vs. sensitive, drought resistant vs. susceptible, and more.

Overall, we investigated the genetics architecture of natural variation in rice salt-tolerance-related traits at the germination stage by GWAS mapping in 295 rice accessions. A total of 79 candidate genes were determined by LD blocks analysis. In addition, by detecting the highly associated variations located inside the genic region that overlapped with the results of LD block analysis, we finally characterized 17 genes that may contribute to salt tolerance during the seed germination stage. The salt tolerance related novel candidate genes would provide important resources for molecular breeding and functional analysis of the salt tolerance during the rice germination.

## 4. Materials and Methods

### 4.1. Materials

A core set of 137 rice accessions and 158 bred varieties from the National Gene Bank of the Rural Development Administration (RDA-Genebank, Korea) [62,63] was re-sequenced in the current study (Supplementary file 1: Table S1). We conducted a field experiment during the rice-growing season at the Kongju National University experimental farm and young leaves from a single plant were collected and immediately kept at −80 °C prior to genomic DNA extraction using the DNeasy Plant Mini Kit (Qiagen, Hilden, Germany). Qualified DNA was sent for the whole genome re-sequencing.

### 4.2. Whole Genome Re-Sequencing and Variation Detection

The genomes of all 295 rice accessions were sequenced with an average coverage of approximately 7.8× on an Illumina HiSeq 2000 or 2500 Sequencing Systems Platform (Illumina Inc., San Diego, CA, USA). Raw reads were aligned against the rice reference genome (IRGSP 1.0) [64] for genotypes calling and only SNPs/indels without the missing value and a minor allele frequency (MAF) > 0.05 and containing genotype calls for all 295 accessions that were used. Lastly, ~1.65 million high-quality SNPs/Indels were obtained and used for the further GWAS [65].

### 4.3. Evaluation of Salt Stress and Phenotyping

We first carried out the pre-screening experiment using 12 randomly selected samples to determine the optimum level of NaCl concentration for the evaluation of salt stress during the germination stage. Seed germination were initially screened by germinating 30 seeds per genotype in petri dishes with two layers of filter papers soaked in two different NaCl concentrations: 200 and 300 mM NaCl. The germination percentage was recorded daily for 10 days. At the concentration of 300 mM NaCl, seeds hardly germinated and the seedlings did not grow out enough to be able to measure root and shoot length. Therefore, in this study, we used 0 mM NaCl (non-stress) and 200 mM NaCl (salt stress) for phenotyping all 295 accessions.

The following experiments were performed in petri dishes containing two-layered filter paper. Thirty seeds were first washed in water, then sterilized in 1% sodium hypochlorite solution for 10 min, and washed three times in deionized distilled water. Thereafter, seeds of each accession were soaked in petri dishes and then incubated at 30 °C with 40% relative humidity. Petri dishes were randomized in an incubator and three replicates of each accession under control and salt conditions (200 mM) were adopted. The solution was replaced every two days to maintain the NaCl concentration and the distilled water volume, respectively. The daily germination seed was measured and filter papers were replaced as necessary. Plumule emergence was taken as an index of germination. The length reached about 2 mm. At the end of day 10, we measured the root length (RL) and shoot length (SL) of the seedlings and the R/S (root/shoot ratio) was also calculated. Based on these experiments, several germination stage-related phenotypes (list below) were calculated and subjected to a GWAS. The mean value of the three biological replicates was calculated and used in the further analysis.

Germination Percentage (GP)

GP was recorded daily for 10 days and was calculated using the formula below.

GP = Number of germinated seeds at 10 days/Total number of seeds tested × 100%

Germination energy (GE)

GE was recorded daily for four days and was calculated using the formula below.

GE = Number of germinated seeds at four days/Total number of seeds tested × 100%

Germination index (GI)

GI was calculated using the formula below.

GI=Σ(Gt/t), where Gt is the number of seeds that germinated on day *t* (Alvarado et al. 1987, and Ruan et al. 2002).

Relative germination energy (R-GE)

R-GE was calculated by using the formula below.

R-GE = GE_200_/GE_control_.

Relative germination index (R-GI)

R-GI was calculated by using the formula below.

R-GI = GI_200_/GI_control._

Relative root length (R-RL)

R-RLwas calculated by using the formula below R-RL = RL_200_/RL_control_.

Relative shoot length (R-SL)

R-SLwas calculated by using the formula below R-SL = SL_200_/SL_control_.

Relative R/S (R-R/S)

R-R/S was calculated by using the formula below R-R/S = R/S_200_/R/S_control_.

### 4.4. Principal Components and GWAS Analysis

Principal components analysis (PCA) of the genotype with ~1.65 million SNPs/indels and four main salt-tolerance-related phenotypes: R-GE, R-GI, R-RL, and R-SL was conducted using GAPIT and Trait Analysis by Association, Evolution and Linkage (TASSEL) 5.0 [66]. Principal component analyses (PCA) in genotypic and phenotypic were also performed using GAPIT and TASSEL 5 [66].

GWAS was performed in the GAPIT package (Genome Association and Prediction Integrated Tool) in which an advanced kinship clustering algorithm was implemented [38]. Only SNPs with adjusted *p*-values < 0.05 were considered significantly associated. Gene loci containing the SNPs with significantly associated peaks in the Manhattan plot of the GWAS result were considered to be candidate genes related to salt tolerance.

### 4.5. Linkage Disequilibrium (LD) Block, Haplotype Analysis, and Expression Analysis

**LD analysis.** LD analysis was calculated using TASSEL 5 [66] based on the high-quality variations (with neither missing genotype calls over all accessions nor MAF < 0.05) in a 400 kb range determined by the most closely associated SNP/indel. An LD block was recognized when the top 95% confidence intervals of the D’ value exceeded 0.98 and the lower bounds exceeded 0.70 [67]. Loci with significant variations harbored by LD blocks were then defined as the candidate genes.

**Haplotype analysis.** With about 7.3× depth of genome coverage, we constructed the haplotyping of the identified candidate genes. Nucleotide polymorphisms on the target genes were captured according to the rice reference genome (IRGSP 1.0). The orthologous genes of the target candidate genes in several other plants were provided by Ensembl Plants (http://plants.ensembl.org). Alignments of orthologous gene sequences were conducted using Geneious (http://www.geneious.com) [68]. In addition, the TCS [69] haplotype network was conducted by PopART v 1.7 [70].

**Gene expression analysis by qRT-PCR.** Germinated seeds with shoot and root after 10 days in control (H_2_O) and salt (200 mM NaCl) conditions were collected and used for expression analysis. Total RNA was prepared using an RNA extraction kit (Qiagen, Hilden, Germany). cDNA was synthesized according to the manufacturer’s instructions using the PrimeScript^TM^ RT reagent Kit (TaKaRa, Shiga, Japan). Real-time PCR was carried out using the SYBR Green method with the primers of Os*MADS31* (MADS-F: TGGCTTCACTGACTCTGCAA, MADS-R: TACATACCCGGCTGTGCATC). Relative expression levels were calculated using the 2^−ΔΔ*C*T^ method [71] with *Ubiquitin 5* (*UBQ 5*) as the internal control [72] under three replicated tests.

## Figures and Tables

**Table 1:** Descriptive statistics for the traits in the control and salt-treated (200 mM NaCl) rice accessions.

Trait	Salinity Level (NaCl/mM)	Mean ± SD ^a^	Range	Median	IQR ^b^
GP	0	0.97 ± 0.06	0.47–1.00	0.99	0.97–1.00
	200	0.87 ± 0.18	0.14–1.00	0.94	0.83–0.98
GE	0	0.95 ± 0.08	0.37–1.00	0.98	0.94–0.99
	200	0.25 ± 0.29	0–0.97	0.13	0.03–0.39
GI	0	47.66 ± 6.71	18.33–57.87	47.55	43.40–53.44
	200	20.66 ± 8.53	1.51–43.72	21.05	15.48–26.03
SL	0	6.07 ± 1.38	3.25–12.62	5.82	5.08–6.90
	200	1.45 ± 0.63	0.38–3.87	1.29	1.03–1.76
RL	0	11.28 ± 2.89	4.28–18.89	11.11	9.46–12.81
	200	0.69 ± 0.49	0.013–3.44	0.55	0.39–0.87

^a^ Standard deviation. ^b^ Interquartile range. GP: Germination percentage. GE: Germination energy. GI: Germination index. SL: shoot length. RL: root length.

**Table 2:** Pearson correlation coefficients among traits under control and salt stress (200 mM NaCl) conditions.

	Trait	GP	GE	GI	SL	RL
**Control**	GP					
	GE	0.946 ***				
	GI	0.624 ***	0.710 ***			
	SL	−0.001 ns	0.037 ns	0.168 **		
	RL	0.073 ns	0.087 ns	0.110 ns	0.254 ***	
**200 mM**	GP					
	GE	0.394 ***				
	GI	0.758 ***	0.849 ***			
	SL	0.439 ***	0.738 ***	0.725 ***		
	RL	0.357 ***	0.618 ***	0.594 ***	0.712 ***	

GP: germination percentage. GE: germination energy. GI: germination index. SL: shoot length. RL: root length. *, **, ***, ns: significant at the 0.05, 0.01, and 0.001 probability level and not significant, respectively.

**Table 3:** Candidate genes with highly associated signals in the coding region that overlapped with the GWAS and LD analysis.

Chr_Pos ^a^	Trait	*p*-Value	FDR ^b^	Gene ID	Description
chr02_19605493	R/S	1.72 × 10^−7^	0.00779	Os02g0532500	Germin family protein, Germin-like protein 2-4
				Os02g0532900	Glycoside hydrolase family 17 protein
				Os02g0533300	Carbonic anhydrase, CAH1-like domain, containing protein
				Os02g0533800	Similar to ATPase inhibitor
chr04_31168058	RL	2.42 × 10^−7^	0.00859	Os04g0612900	Vacuolar ATPase assembly integral membrane protein VMA21-like domain-containing protein
				Os04g0613900	Similar to Potassium transporter 18, OsHAK11
				Os04g0614000	Similar to Peroxisomal 2,4-dienoyl-CoA reductase
				Os04g0614100	MADS-box domain-containing protein, OsMADS31
				Os04g0614600	Similar to Viroid RNA-binding protein, aminotransferase
				Os04g0614500	Pyridoxal phosphate-dependent transferase, major region, subdomain 1 domain-containing protein
				Os04g0615100	Similar to Lecithine cholesterol acyltransferase-like protein
				Os04g0615700	Protein argonaute 2, OsAGO2
chr12_1628276	RL, R/S, R-R/S	2.02 × 10^−7^	0.00859	Os12g0133100	Major facilitator superfamily protein, OsZIFL12
				Os12g0133300	zinc-induced facilitator-like 13, OsZIFL13
				Os12g0133400	4′-phosphopantetheinyl transferase domain-containing protein
				Os12g0133700	Stress-activated protein kinase pathway-regulating phosphatase 1
				Os12g0133800	Similar to Auxin efflux carrier protein, *OsPIN1d*

^a^ The position was based on the annotation data on Os-Nipponbare-Reference-IRGSP-1.0 (RAP-DB, http://rapdb.dna.affrc.go.jp/). ^b^ FDR: False discovery rate. FDR Adjusted *p* values were calculated by GAPIT applying the Benjamini-Hochberg (1995) FDR-controlling procedure. RL: root length. R/S: root/shoot ratio. R-R/S: relative root/shoot ratio.

## Supplementary Materials

The following are available online at http://www.mdpi.com/1422-0067/19/10/3145/s1.

## Abbreviations

GWAS	genome-wide association study
LD	linkage disequilibrium
NGS	next-generation sequencing
SNP	single-nucleotide polymorphism
INDEL	insertion and deletion
MAF	minor allele frequency
RL	root length
SL	shoot length
RS	root/shoot ratio
GP	Germination percentage
GE	Germination energy
GI	Germination index
CMLM	compressed mixed linear model
PCA	Principal component analysis
