# Supplementary material for: A Genome-Wide Association Study Reveals Candidate Genes Related to Salt Tolerance in Rice (Oryza sativa) at the Germination Stage
Source: Int J Mol Sci. 2018 Oct 12;19(10):3145. doi: 10.3390/ijms19103145 (PMC6213974; doi:10.3390/ijms19103145)
Supplement: Supplementary file 1 [file ijms-19-03145-s001.zip › Supplementary file 2 Figure S1 and Figure S2.docx]

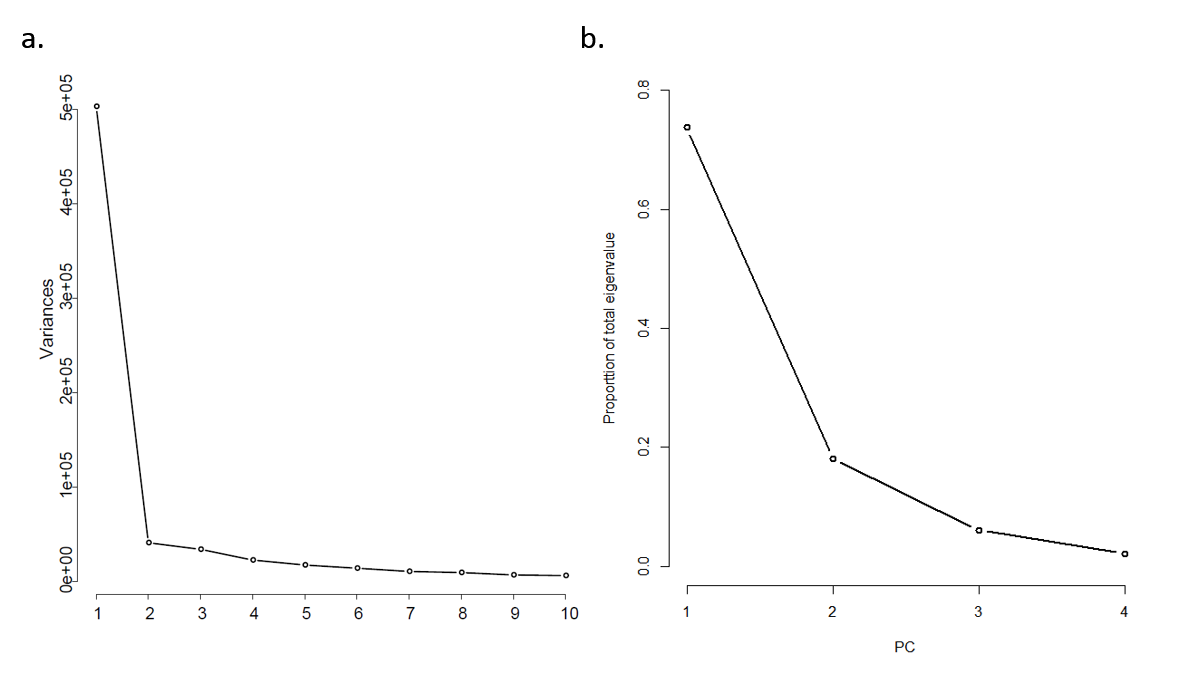


**Fig. S1** Scree plot determining the appropriate number of principal components. The most obvious eigenvalues change in slope in the scree plot occurs at component 2 in both genotype (**a**) and phenotype (**b**), suggesting that PC1 and PC2 can explain most proportion of total eigenvalues. The appropriate number of principal components is two.


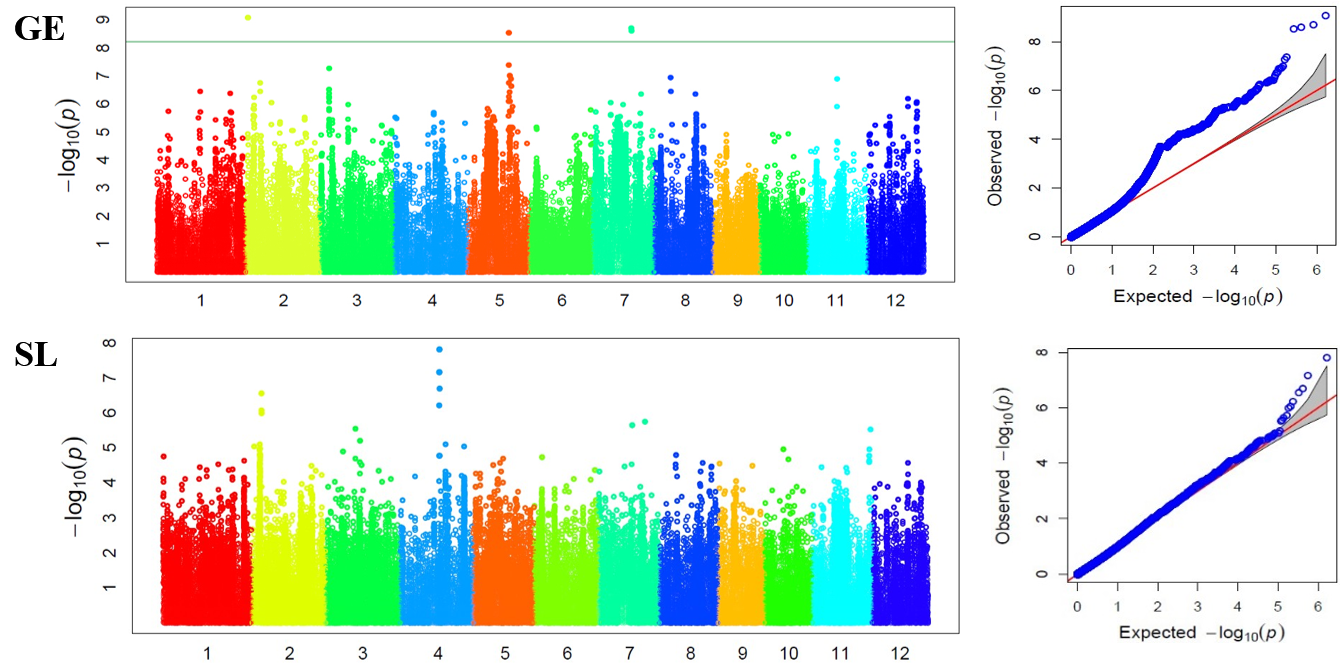

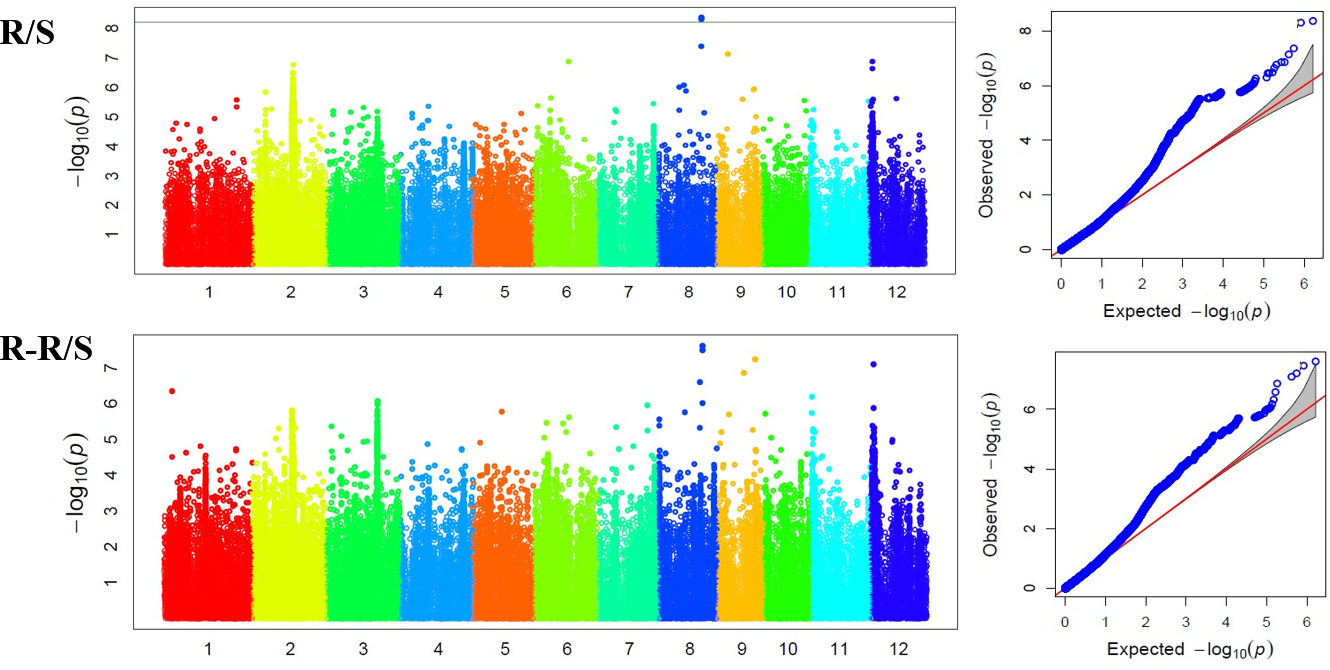


**Fig. S2** Manhattan plots and QQ plots for several salt tolerance phenotypes identified by the genome-wide association study that did not show strong associations. –Log_10_ (*P*) values from a genome-wide scan are plotted against position on each of the 12 chromosomes. GE: Germination Energy; SL: Shoot Length; R/S: root / shoot ratio; R-R/S: relative root / shoot ratio.
